# Generational Advancements in the Transverse Shear Strength Retention of Glass Fiber-Reinforced Polymer Bars in Alkaline and Acidic Environments

**DOI:** 10.3390/polym16192712

**Published:** 2024-09-25

**Authors:** Mesfer M. Al-Zahrani

**Affiliations:** 1 Civil and Environmental Engineering Department, King Fahd University of Petroleum & Minerals, Dhahran 31261, Saudi Arabia; mesferma@kfupm.edu.sa; 2 Interdisciplinary Research Center for Construction and Building Materials (IRC-CBM), King Fahd University of Petroleum & Minerals, Dhahran 31261, Saudi Arabia

**Keywords:** glass fiber-reinforced polymer (GFRP) bars, long-term performance, accelerated aging, artificial neural network, linear regression

## Abstract

In this study, the transverse shear strength (TSS) retention of two types of new-generation glass fiber-reinforced polymer (GFRP) bars, namely ribbed (RB) and sand-coated (SC) bars, was investigated under alkaline, acidic, and marine conditions in both high-temperature and laboratory environments for up to one year. The ribbed GFRP bars exhibited no notable reduction in strength under ambient conditions after 12 months, but under high-temperature conditions (60 °C), they showed TSS reductions of 10.6%, 9.7%, 11.1%, and 10.9% for exposure solutions E1, E2, E3, and E4, respectively. The sand-coated GFRP bars showed slight strength reductions under ambient conditions and moderate reductions under high-temperature conditions (60 °C), with TSS reductions of 22.5%, 29.0%, 13.0%, and 13.7% for the same solutions, highlighting the detrimental effect of high temperatures on the degradation of the resin matrix. Comparative analyses of older-generation ribbed (RB-O1 and RB-O2) and sand-coated (SC-O) GFRP bars exposed to similar conditioning solutions for the same duration were also performed. In addition, linear regression and artificial neural network (ANN) models were developed to predict strength retention. Models developed using linear regression and ANNs achieved coefficients of determination (*R*^2^) of 0.69 and 0.94, respectively, indicating that the ANN model is a more robust tool for predicting the TSS of GFRP bars than is the conventional linear regression model.

## 1. Introduction

The properties of glass fiber-reinforced polymer (GFRP) bars, such as corrosion resistance, superior strength-to-weight ratio as compared to convention steel rebars, good tensile strength, and electrical and magnetic transparency, have led to their wide application in both infrastructure development and the replacement of conventional steel rebars [1,2,3,4,5]. GFRP bars offer several advantages over other fiber-reinforced polymers (FRPs), such as carbon fiber-reinforced polymers (CFRP) and basalt fiber-reinforced polymers (BFRP), particularly in terms of cost-effectiveness and durability [6]. GFRP is significantly more affordable than CFRP, making it a viable choice for large-scale projects where cost constraints are critical, without substantially sacrificing performance. It is highly durable in harsh environments, offering excellent resistance to moisture, and chemicals, which makes it ideal for long-term use in infrastructure projects.

Although extensive research and field investigations have led to the standardization of GFRP bars as reinforcements in concrete structural members [7,8,9], durability studies are necessary to investigate the durability performance of GFRP bars in aggressive environments, such as alkaline, acidic, and marine conditions, which can significantly affect their long-term performance.

Elevated temperatures and harsh environmental conditions are key factors that significantly weaken the strength of GFRP bars. The polymeric resins forming the matrix generally have a glass transition temperature (*T_g_*) ranging from 65 to 120 °C [10,11]. When the exposure temperature approaches this range, the resin begins to soften, which causes a significant reduction in the composite bonding between the glass fibers and matrix, which in turn compromises the overall structural integrity of the composite, and leads to diminished load-transfer efficiency and significant degradation in mechanical performance under stress [12].

The transverse shear strength (TSS) of GFRP bars is an important mechanical property that determines the ability of composite bars to resist shear forces acting perpendicular to the longitudinal axis, which is essential in reinforcement applications in which the bars experience complex stress states [13]. While tensile strength is vital for GFRP bars due to their primary function in resisting axial loads, the significance of transverse shear strength cannot be overlooked. In concrete structures, GFRP bars are subjected not only to tensile forces but to shear stresses, especially in regions of high bending or near support zones where shear forces are prominent. The study of TSS is crucial to ensure that GFRP bars maintain their integrity under these multi-directional stress conditions. A reduction in transverse shear strength can lead to shear failure, which severely affects the structural performance and safety of reinforced systems. Understanding the resilience of GFRP bars in terms of their TSS after prolonged exposure to such conditions is essential for predicting their service life and ensuring the safety and reliability of reinforced concrete structures.

The deterioration of the mechanical strength of GFRP bars exposed to harsh environments is often attributed to phenomena such as hydrolysis of the resin matrix [14] or leaching of glass fibers [15], which result in swelling and microcracking at the glass-matrix interfaces [16]. Water molecules penetrate the resin matrix, leading to hydrolysis reactions which results in the breaking of polymer chains and reducing the bonding force between the molecules [16]. Leaching of glass fibers occurs when glass dissolves in water, causing alkalis to be extracted from the glass structure, generating hydroxides, raising the pH above 9, which leads to the degradation of the Si networks (Si–O–Si and Si–O–Na/K) in the glass. The process produces Si–OH, a gel-like product that is less dense than the original glass and facilitates the transport of water and alkalis, accelerating fiber degradation [17,18,19,20].

The American Concrete Institute (ACI) provisions, such as ACI 440.3R-12 [21], and researchers [22,23,24,25,26] have both reported the use of accelerated aging techniques to simulate the long-term durability of GFRP bars. These techniques involve the immersion of bars in solutions that simulate the alkalinity of the pore water in Portland-cement concrete and are typically applied at a temperature of 60 °C [15]. Microstructural investigations are generally performed on GFRP bars subjected to aggressive environments to assess the degradation mechanisms and changes in fiber–matrix interactions, and to evaluate the long-term durability and performance of the bars [3,27,28,29,30,31,32].

Sawpan [33] investigated the long-term durability of GFRP rebars exposed to seawater at different temperatures (23, 55, and 75 °C) over durations of 8 and 20 months. The study revealed that the TSS increased under dry conditions at elevated temperatures but decreased when conditioned in seawater, particularly at higher temperatures. The glass transition temperatures (*T_g_*) of the bars remained relatively stable, indicating that no significant polymer degradation occurred. Morales et al. [34] investigated the long-term durability of GFRP bars embedded in concrete produced from seawater. Investigations were conducted on the mechanical and physical properties of bars exposed to typical field conditions and accelerated aging in seawater at 60 °C for up to 24 months. Major findings revealed a significant reduction in tensile strength under aggressive conditions, with a predicted long-term retention of approximately 72% in seawater environments.

Yu et al. [23] studied the effects of various alkalinity levels on the durability of basalt fiber-reinforced polymer (BFRP) bars subjected to a simulated seawater sea-sand concrete (SWSSC) environment. Reducing the alkalinity of the SWSSC environment significantly mitigated moisture uptake and shear strength loss in the BFRP bars. Lowering the pH from 13.2 to 10.1 notably decreased moisture uptake, lowering it from 5.47% to 0.14%, and maintained the transverse and interlaminar shear strength retentions.

Machine learning algorithms are extensively used in research because of their ability to generate accurate predictions without the need to formulate explicit governing equations. This capability is particularly valuable for addressing complex, nonlinear problems, as conventional methods are often incapable of properly capturing the underlying complexities of such problems [35,36,37]. Artificial neural networks (ANNs) are computational models used in machine learning that mimic the structure and capability of the human brain in processing highly complex data and recognizing patterns [38,39]. ANNs comprise layers of interconnected nodes or neurons associated with weights and biases that are adjusted during the learning process. The ability of ANNs to effectively manage nonlinear relationships and large datasets makes them particularly suitable for applications such as image recognition, natural language processing, and predictive modeling. Although numerous researchers have employed machine learning techniques to predict concrete strength with good accuracy [35,40,41,42,43], limited applications [15] of machine learning models exist for the TSS prediction of GFRP bars in the literature.

In this study, the transverse shear strength (TSS) retention of new-generation GFRP bars, namely, ribbed (RB) and sand-coated (SC) bars, were investigated when exposed to accelerated aging in alkaline, acidic, and marine environments under both high temperature (60 °C) and ambient laboratory conditions (~20 °C) for up to one year. This study compared the results from new-generation bars with older-generation ribbed (RB-O1 and RB-O2) and sand-coated (SC-O) GFRP bars subjected to similar exposure conditions. The results indicate that new-generation GFRP bars demonstrate superior resilience to harsh environmental conditions, making them promising alternatives to steel reinforcements in corrosive environments. In addition, a predictive model was developed using both linear regression and an ANN. The ANN model proved to be a more robust tool for predicting TSS retention in GFRP bars than did the conventional linear regression model.

## 2. Materials and Methods

### 2.1. Materials

#### GFRP Bars

**Figure 1:**
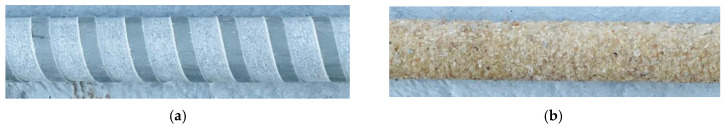
GFRP bar types. (**a**) Ribbed GFRP (RB). (**b**) Sand-coated GFRP (SC).

Figure 1 shows the durability performances of two types of new-generation GFRP bars, namely, ribbed-type (RB) and sand-coated-type (SC) bars. The surface of bar RB was characterized by prominent, evenly spaced spiral ridges running along its length and had a texture with evenly distributed grainy sand particles. Bar RB had a slightly larger diameter (13.71 mm) than did bar SC (13.02 mm). The ultimate tensile strengths of RB and SC were determined to be 956.4 MPa (with an elastic modulus of 49.5 GPa) and 1029.6 MPa (with an elastic modulus of 43.6 GPa), respectively. Table 1 lists the detailed technical properties of the GFRP bars.

**Figure 2:**
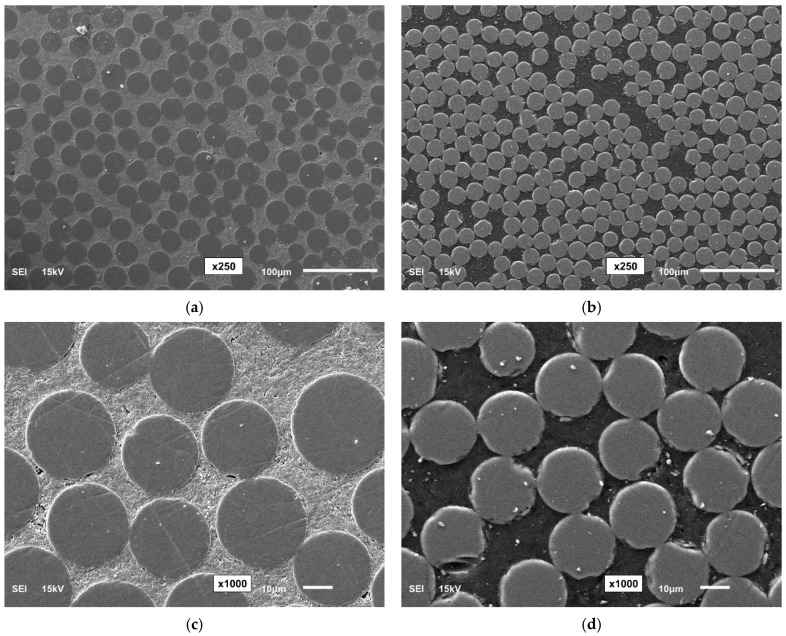

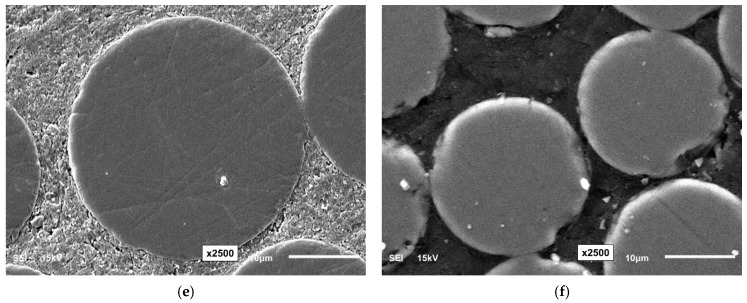
SEM micrographs of the GFRP bars. (**a**) RB (250× magnification), (**b**) SC (250× magnification), (**c**) RB (1000× magnification), (**d**) SC (1000× magnification), (**e**) RB (2500× magnification), (**f**) SC (2500× magnification).

Figure 2 shows SEM micrographs of the bars at magnifications of 250×, 1000×, and 2500× using JEOL SEM (Model JSM6610LV, JEOL Ltd., Tokyo, Japan) equipment. To obtain the micrographs, the GFRP bar specimens were cut into 20 mm pieces using a fine-toothed hacksaw, then cold-mounted in epoxy resin and cured for 24 h. The cross-sections were polished with sandpaper of increasing grits (150–1200), followed by diamond pastes (9 and 3 µm). To ensure conductivity, a 10 nm gold layer was sputter-coated [15].

### 2.2. Exposure Conditions

GFRP rebars embedded in concrete are subjected to a highly alkaline environment, with pH levels ranging from 12.4 to 13.7 [21,44]. The conditioning solution E1 was prepared according to ACI 440.3R-12 [21] by dissolving 118.5 g of Ca(OH)_2_, 0.9 g of NaOH, and 4.2 g of KOH in 1 L of deionized water, achieving a pH value between 12.6 and 13. Additionally, solution E2 was prepared by introducing 3% sodium chloride into E1 to simulate a seawater environment. Both E1 and E2 reached a pH of 13.4. To simulate acid exposure, solution E3, with a pH of 3.1, was prepared using acetic acid (0.6% CH_3_COOH) [15]. To evaluate the performance of the GFRP bars under high-humidity conditions, pH-neutral tap water was used as solution E4.

**Figure 3:**
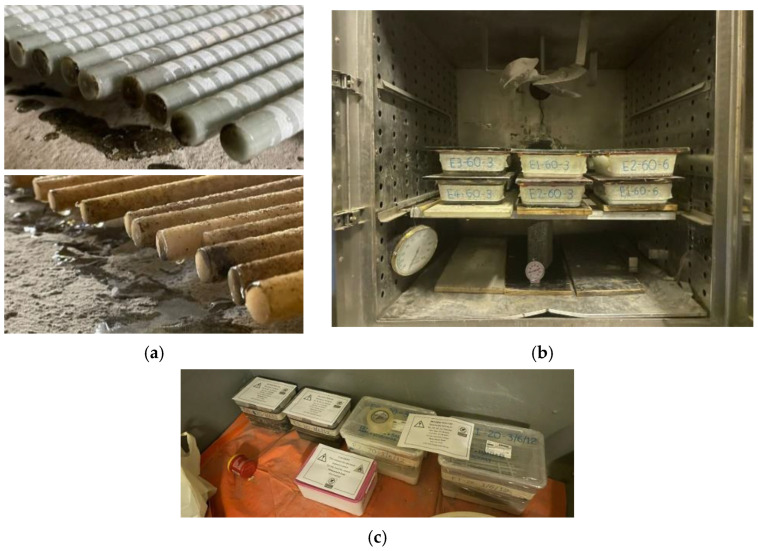
Sample preparation and exposure environments. (**a**) End-coatings on bars. (**b**) High-temperature exposure. (**c**) Lab exposure.

To investigate the effect of temperature on the durability of the samples, conditioning was carried out at approximately 20 °C, representing a controlled indoor laboratory environment, and at 60 °C, simulating high-temperature conditions [15,33,45,46]. First, the GFRP bars were cut into lengths of 300 mm, and their ends were sealed with epoxy resin to prevent the conditioning solutions from penetrating the matrix through the cut surfaces [21], as shown in Figure 3a. According to the manufacturer, the epoxy is expected to achieve a compressive strength of 83 MPa after 7 days of curing. Therefore, the end-coated specimens were stored in a contamination-free room for more than 7 days to ensure full curing. The prepared specimens were then arranged in containers, filled with the corresponding conditioning solutions, and sealed with a high-temperature gasket silicone to minimize moisture loss. These containers were subsequently stored in either an environmental chamber for high-temperature exposure (Figure 3b) or an ambient laboratory environment (Figure 3c).

### 2.3. GFRP Strength Retention

#### 2.3.1. Transverse Shear Strength

**Figure 4:**
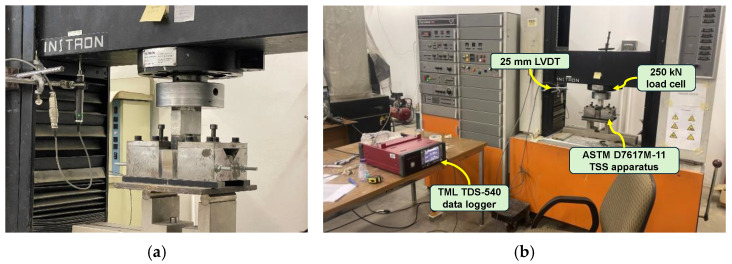
Mechanical strength testing of GFRP bars. (**a**) ASTM D7617M-11 [47] apparatus. (**b**) Full test setup with instrumentation.

The TSS values of the GFRP bars were determined according to ASTM D7617M-11 [47]. The testing apparatus (Figure 4a) was fabricated according to standard specifications. The apparatus comprised two V-shaped seats positioned on bar specimens. A setup with one upper blade and two lower blades was designed to create two shear planes during loading. Blades with 13 mm slots were fabricated to accommodate the specimens tested in the experimental program. GFRP bars cut to a length of 225 mm were placed in a V-shaped bar seat and securely fastened with screws.

The TSS values of the GFRP bars were calculated using the ASTM D7617M-11 equation [47], as shown in Equation (1), where τ is the TSS, $P_{s}$ is the peak failure load, and A is the cross-sectional area of the specimen:

(1) $$\tau \;=\frac{P_{s}}{A}$$

#### 2.3.2. Instrumentation

The GFRP bar specimens were subjected to displacement-controlled loading at a rate of 1 mm/min. The applied load was measured using an Instron load cell with a capacity of 250 kN, as shown in Figure 4b. The displacement of the crosshead was recorded simultaneously using a linear variable differential transducer (LVDT). Both the load cell and the LVDT were connected to a Tokyo Sokki Kenkyujo TDS 540 data logger for data acquisition.

### 2.4. Test Matrix

**Figure 5:**
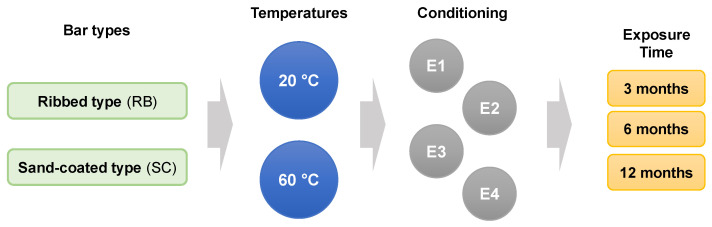
Test matrix (E1: alkaline; E2: alkaline and salt; E3: acidic; and E4: water).

The experimental plan was structured to assess all possible exposure combinations discussed in the previous section (as illustrated in Figure 5), necessitating the testing of 250 GFRP bar specimens for transverse shear strength. The transverse shear strength of each combination was determined using five replicates, following the ASTM D7617M-11 guidelines [47]. In addition, unconditioned control specimens were tested to establish the baseline transverse shear strength. Table 2 presents the tabulated form of the test matrix along with the name of each combination.

### 2.5. Strength Prediction Models

Linear regression models the relationship between a dependent variable (response) and one or more independent variables (predictors) by fitting a linear equation to the observed data [15,48]. The basic mathematical expression for the linear regression is given by Equation (2), where y is the dependent variable, $x_{1},$ $x_{2},\;\ldots ,\;x_{n}$ are the independent variables,$\;\beta_{1},$ $\beta_{2},\;\ldots ,\;\beta_{n}$ are the fitting parameters, and ϵ is the error term:

(2) $$y\;=\;\beta_{0}+\beta_{1}x_{1}+\beta_{2}x_{2}+\cdots +\beta_{n}x_{n}+\epsilon$$

**Figure 6:**
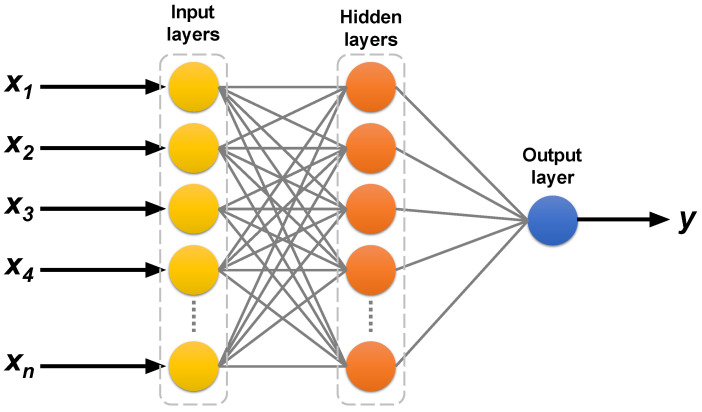
ANN architecture.

Artificial neural networks (ANNs) are computational models inspired by the structure and function of the human brain, and are primarily used in applications involving machine learning and artificial intelligence [49]. ANNs consist of interconnected layers of nodes, also referred to as “neurons,” that work together to perform data processing, pattern recognition, decision-making, and prediction tasks [39]. As shown in Figure 6, the typical architecture of an ANN comprises an input layer, several hidden layers, and an output layer. Each neuron in a layer is connected to neurons in the subsequent layer, and each connection is assigned a weight that is adjusted as the network learns from the data.

The process of modeling in an artificial neural network starts with the training phase, in which portions of the input and output data are utilized by the model to learn patterns and trends from the input data. This is followed by a validation phase, during which the trained model is assessed on a different subset of data [50]. Finally, in the testing phase, a separate test subset is used to evaluate the performance of the fully trained model objectively. In this study, the neural network toolbox in MATLAB was used to create a feedforward backpropagation model using the Levenberg–Marquardt algorithm. The input dataset was divided into 70%, 15%, and 15% subsets for training, validation, and testing, respectively.

## 3. Results and Discussion

### 3.1. TSS–Displacement Response

#### 3.1.1. Control Bars

**Figure 7:**
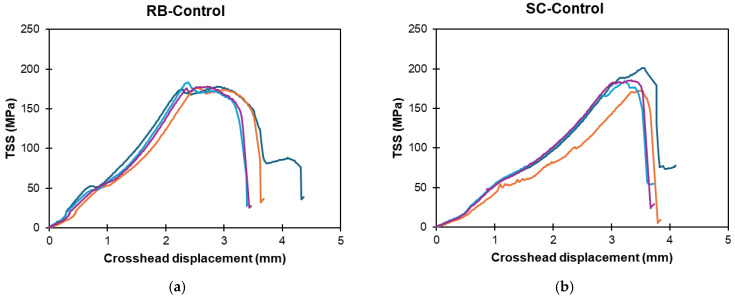
Transverse shear stress versus crosshead displacement of control RB and SC bars. (**a**) Ribbed-type GFRP (RB). (**b**) Sand-coated type GFRP. (Note: multiple lines indicate replicates).

Figure 7a,b present the TSS displacement responses of the RB and SC bar replicates, respectively. The ribbed-type (RB) bars exhibited an initial linear increase in transverse shear stress with increasing crosshead displacement, reaching a mean TSS of approximately 180.1 MPa. Beyond this peak, the TSS sharply declined, indicating a sudden loss in the transverse shear capacity. The sand-coated (SC) bars also showed a linear increase with crosshead displacement, reaching an average peak TSS of approximately 185.1 MPa. The SC bars, with their rough surface, exhibited slightly better mechanical strength, which is likely due to a more uniform distribution of stresses, reducing stress concentrations that could lead to premature failure. The importance of these sand coatings becomes more evident in SC bars when sand particles are ejected under harsher exposure conditions, as presented later in the study.

#### 3.1.2. Conditioned Bars

**Figure 8:**
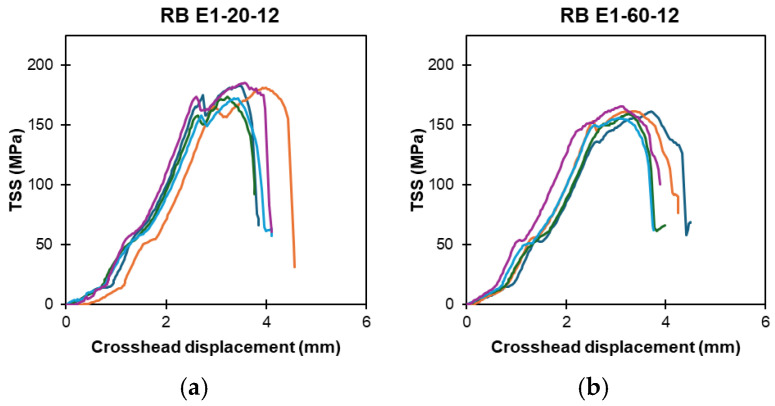

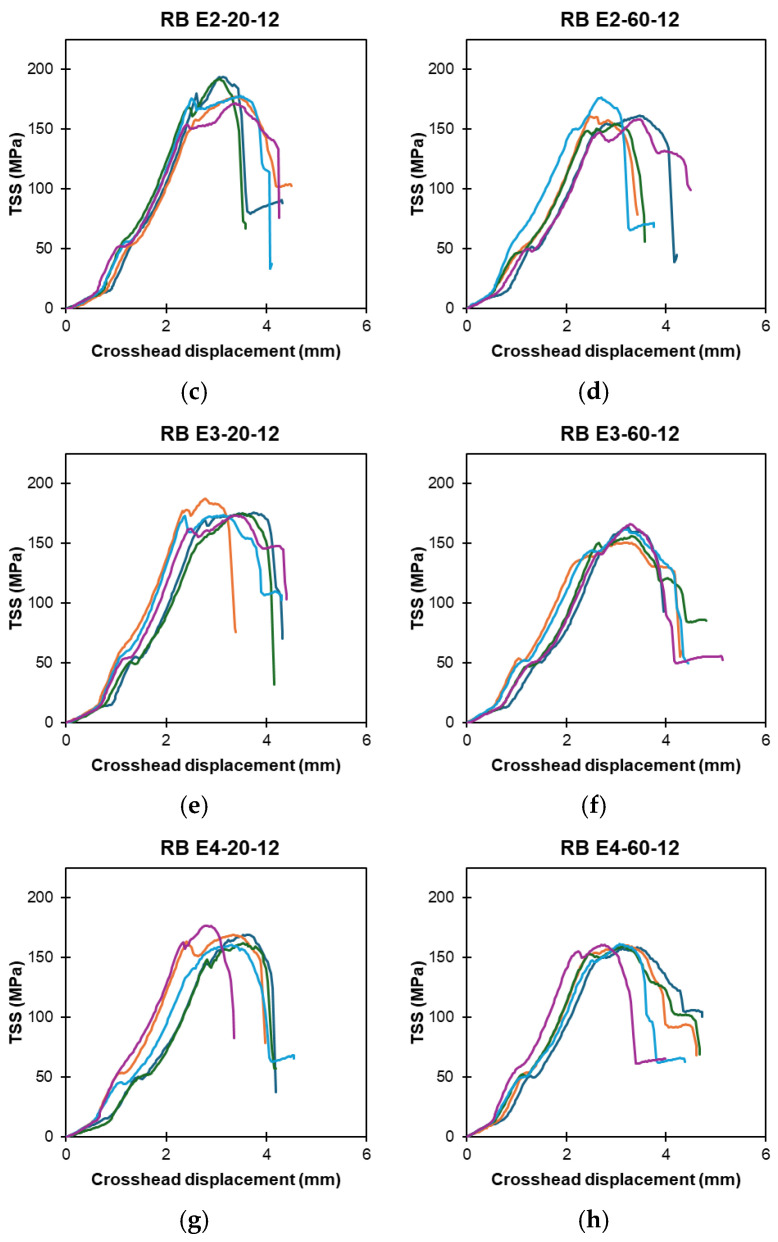
Ribbed-type (RB) bars exposed to high-temperature: TSS versus displacement response at 6 and 12 months (notation: exposure solution-temperature-exposure duration). (**a**) RB: E1-20-12. (**b**) RB: E1-60-12. (**c**) RB: E2-20-12. (**d**) RB: E2-60-12. (**e**) RB: E3-20-12. (**f**) RB: E3-60-12. (**g**) RB: E4-20-12. (**h**) RB: E4-60-12. (Note: multiple lines indicate replicates).

**Figure 9:**
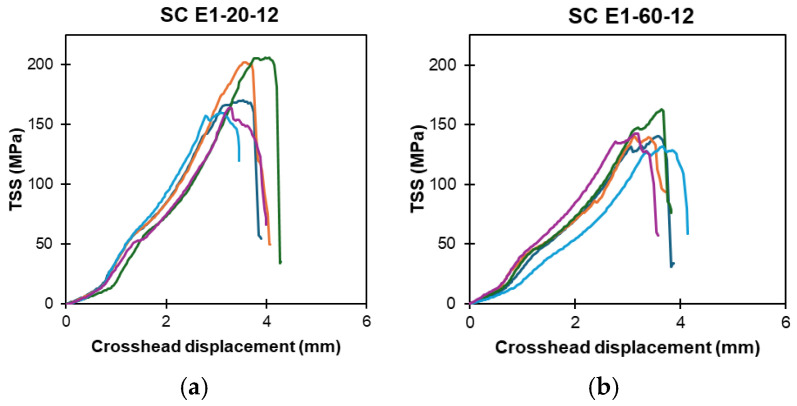

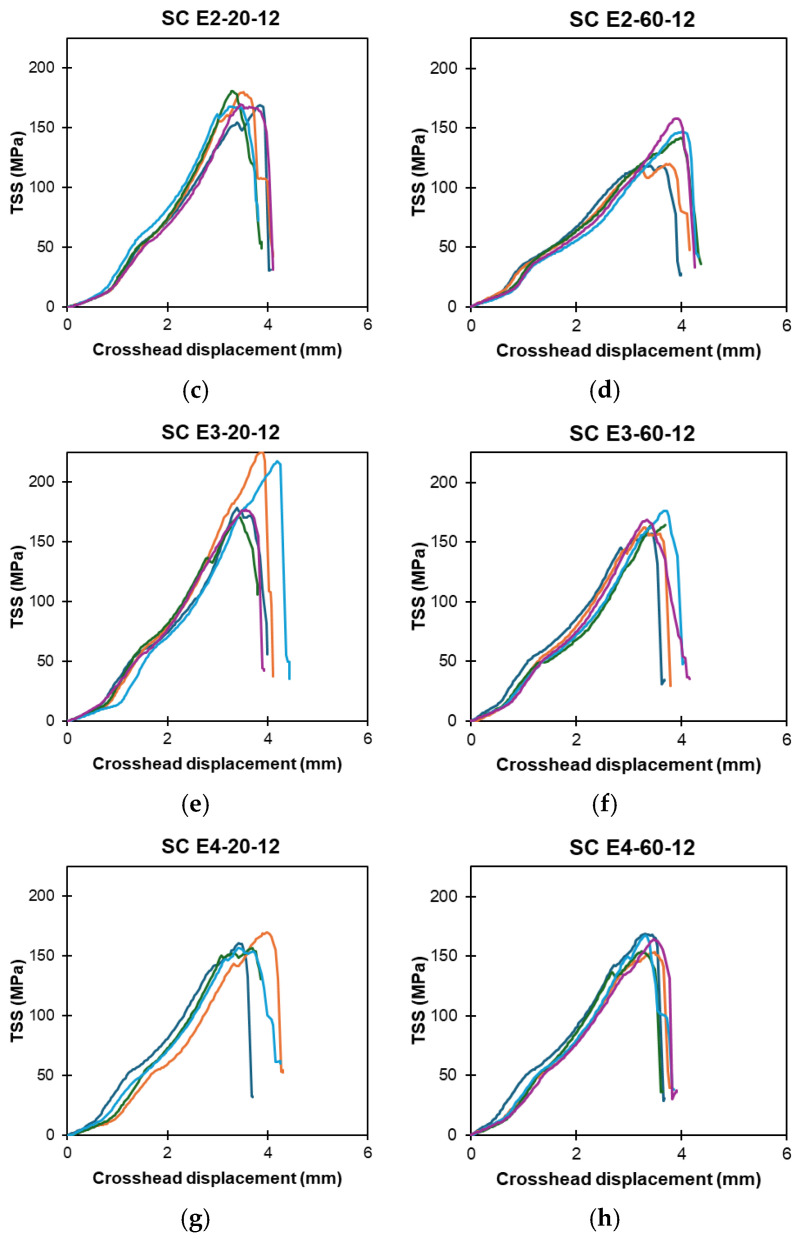
Sand-coated type (SC) bars exposed to high-temperature: TSS versus displacement response at 6 and 12 months (notation: exposure solution-temperature-exposure duration). (**a**) SC: E1-20-12. (**b**) SC: E1-60-12. (**c**) SC: E2-20-12. (**d**) SC: E2-60-12. (**e**) SC: E3-20-12. (**f**) SC: E3-60-12. (**g**) SC: E4-20-12. (**h**) SC: E4-60-12. (Note: multiple lines indicate replicates).

Figure 8a–h present the TSS displacement of a ribbed-type GFRP bar, denoted by RB, that was subjected to exposure solutions E1, E2, E3, and E4 in high-temperature regimes. Similarly, Figure 9a–h illustrate the TSS displacement of the sand-coated GFRP bar specimens, denoted by SC, that were exposed to solutions E1, E2, E3, and E4 in high-temperature regimes.

### 3.2. Effect of Exposure Solution on TSS

**Figure 10:**
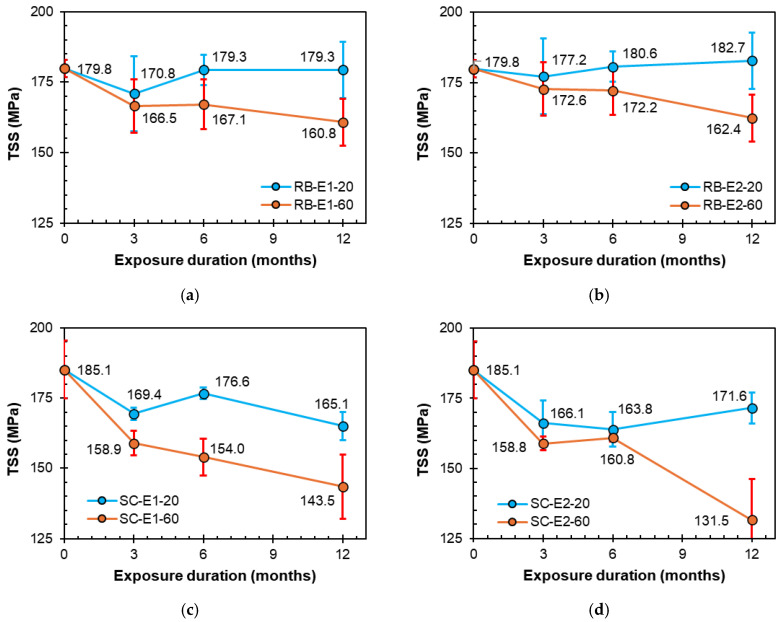
Strength degradation owing to E1 (alkaline) and E2 (alkaline and salt) conditionings. (**a**) RB bars exposed to E1 (alkaline). (**b**) RB bars exposed to E2 (alkaline and salt). (**c**) SC bars exposed to E1 (alkaline). (**d**) SC bars exposed to E2 (alkaline and salt).

This section analyzes the effects of various exposure conditions on the TSS retention of the GFRP tested in this study. Figure 10a,b illustrate the evolution of the ribbed RB bars under E1 (alkaline) and E2 (alkaline and salt) conditions, respectively, over different exposure durations (3, 6, and 12 months). Under ambient laboratory conditions (20 °C), the bars were found to be resilient to the alkaline solutions, and they maintained their original TSS for 12 months. However, the TSS values of the bars were found to decline with time at the high temperature of 60 °C. After 12 months at 60 °C, the TSS values were 160.8 and 162.4 MPa, exhibiting reductions of 10.6% and 9.7% when exposed to the E1 and E2 solutions, respectively.

Figure 10c,d show the variation in the ribbed RB bars under E1 (alkaline) and E2 (alkaline and salt) conditions, respectively, over exposure durations of 3, 6, and 12 months. Unlike the ribbed RB bars, the sand-coated SC bars were adversely affected by the exposure solutions under laboratory temperature conditions. After 12 months at the laboratory temperature, the TSS values were 165.1 and 171.6 MPa, exhibiting reductions of 10.8% and 7.3% when exposed to the E1 and E2 solutions, respectively. After 12 months at 60 °C, the TSS reduced by 22.5% and 29.0% when exposed to the E1 and E2 solutions, respectively.

**Figure 11:**
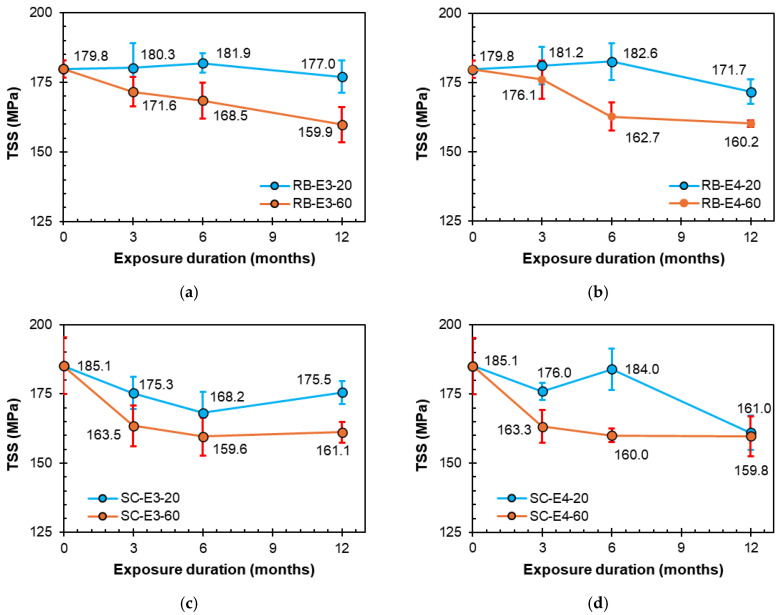
Strength degradation owing to E3 (acidic) and E4 (water) conditions. (**a**) RB bars exposed to E3 (acid). (**b**) RB bars exposed to E4 (water). (**c**) SC bars exposed to E3 (acid). (**d**) SC bars exposed to E4 (water).

Figure 11a,b present the effects of the E3 (acid) and E4 (water) conditions, respectively, on the TSS retention of RB bars over different exposure durations of 3, 6, and 12 months. Under laboratory conditions (20 °C), the bars were found to show slight reductions in strength after 12 months. However, the strength of the bars was found to decline with time upon exposure to the high temperature of 60 °C. After 12 months at 60 °C, the TSS values were 159.9 and 160.2 MPa, respectively, exhibiting reductions of 11.1% and 10.9% when exposed to the E3 and E4 solutions, respectively.

Figure 11c,d show the reduction in the TSS values of the sand-coated SC bars under the E3 (acid) and E4 (water) conditions, respectively, over exposure durations of 3, 6, and 12 months. Unlike the ribbed RB bars, the sand-coated SC bars were adversely affected by the exposure solutions under laboratory temperature conditions. After 12 months at the laboratory temperature, the TSS values were 175.5 and 161.0 MPa, respectively, exhibiting reductions of 5.2% and 13.0% when exposed to the E3 and E4 solutions, respectively. After 12 months at 60 °C, the TSS values declined by 13.0% and 13.7% when exposed to the E3 and E4 solutions, respectively. This significant decline in mechanical strength of GFRP bars exposed to harsh environments is often due to phenomena such as hydrolysis of the resin matrix [14] or leaching of glass fibers [15], resulting in the damage of glass-matrix interfaces [16].

The observed increase in transverse shear strength with immersion time in some cases, shown in Figure 10 and Figure 11, may be attributed to the post-curing effect of the resin matrix. Post-curing occurs when the resin matrix continues to cure over time, even after the initial manufacturing process, which allows the polymer chains to further cross-link, improving the overall mechanical properties of the matrix, such as stiffness and strength [51].

**Figure 12:**
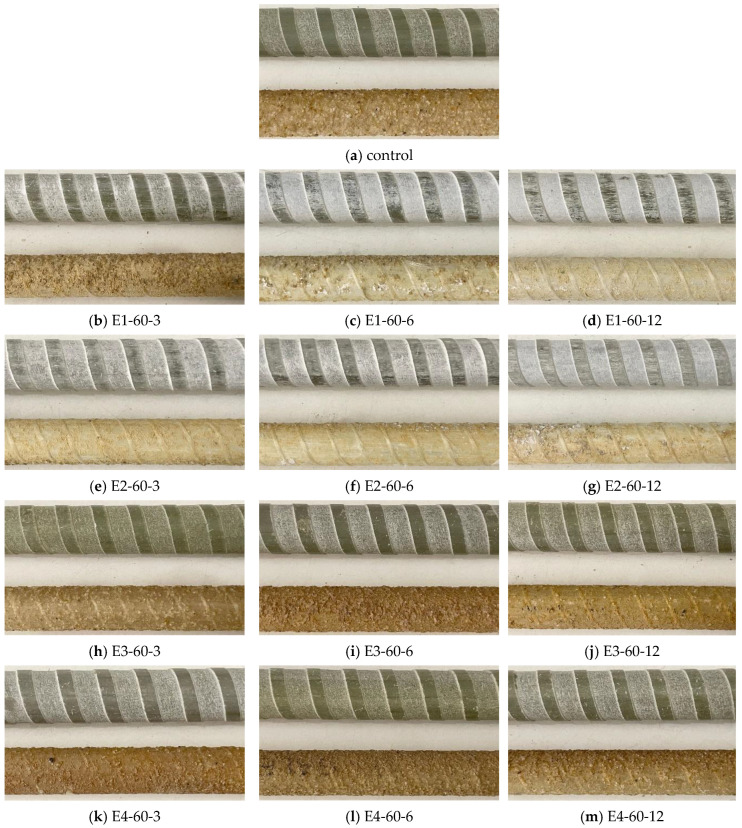
Conditions of the surfaces of high-temperature exposed GFRP bars (E1: alkaline; E2: alkaline and salt; E3: acid; and E4: water); (notation: exposure solution-temperature-time).

Figure 12 shows the surface textures of both the control and high-temperature-exposed GFRP bars. High-temperature exposure led to the detachment of the sand coatings from the bars, which exposed the polymeric matrix to aggressive species and caused greater strength reductions in the SC bars after exposure.

### 3.3. Comparison with Older-Generation Bars

#### 3.3.1. Reference Study

This study investigated the effects of various accelerated aging conditions on two types of new-generation GFRP bars. Hence, it is interesting to compare the performances of these bars with those of older-generation bars subjected to similar exposure conditions. Thus, this study compares the TSS results with those of an investigation conducted by the authors’ research team to determine the TSS retention of three types of GFRP bars that have been manufactured for more than two decades [15]. The reference study by Fasil and Al-Zahrani [15] used three types of GFRP bars, namely, ribbed type 1 (RB-O1), ribbed type 2 (RB-O2), and sand-coated (SC-O), under four types of exposure conditions: alkaline (0.6N KOH + 0.2N NaOH. Ca(OH)_2_), alkaline with 3% NaCl, acidic (0.6% CH_3_COOH), and water. The older generation bars investigated in the referenced study were manufactured over 25 years ago, making them ideal candidates for observing advancements in newer generations of GFRP bars. As with this study, the specimens were exposed to two different temperature regimes: the ambient laboratory temperature and a high temperature of 60 °C.

**Figure 13:**
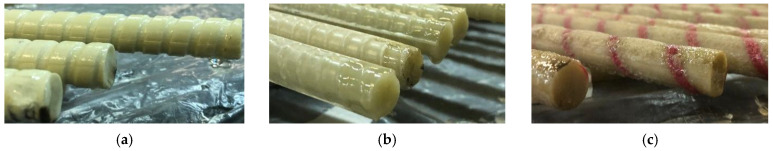
Surface features of the older-generation GFRP bars. (**a**) Ribbed type-1 (RB-O1). (**b**) Ribbed type-2 (RB-O2). (**c**) sand coated-type (SC-O).

**Figure 14:**
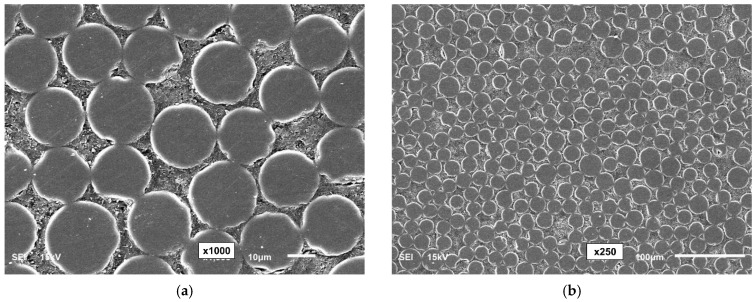

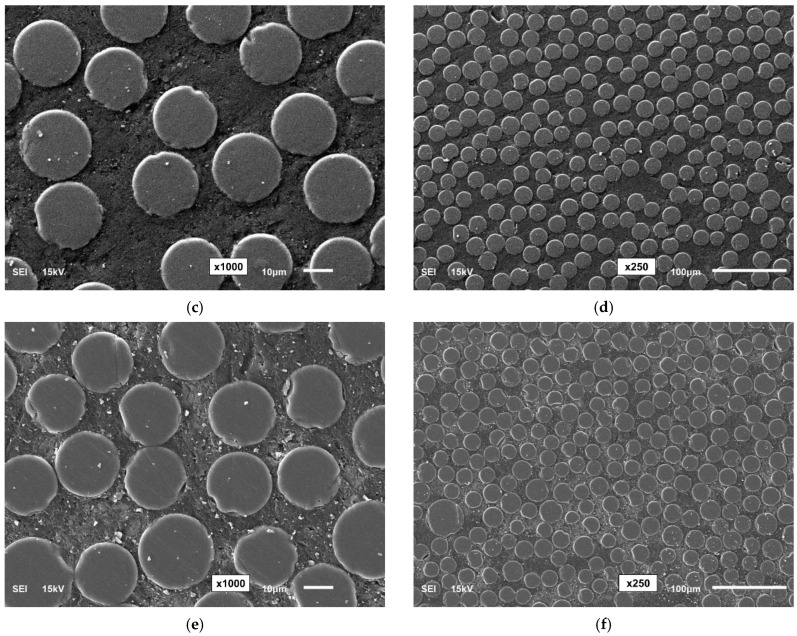
SEM micrographs of RB-O1, RB-O2, and SC-O. (**a**) RB-O1 (at 1000×). (**b**) RB-O1 (at 250×). (**c**) RB-O2 (at 1000×). (**d**) RB-O2 (at 250×). (**e**) SC-O (at 1000×). (**f**) SC-O (at 250×).

Figure 13a–c show the surface characteristics of the RB-O1, RB-O2, and SC-O bars. Table 3 lists the properties of the older-generation GFRP bars, including their diameters, glass fibers, matrix types, and transverse shear strength. Figure 14 presents SEM micrographs (at 1000× and 250× magnifications) of the GFRP bars.

#### 3.3.2. Effect of Conditioning

**Figure 15:**
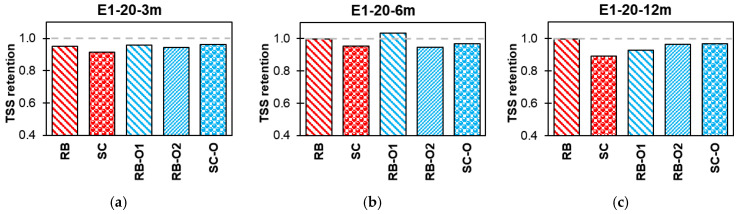

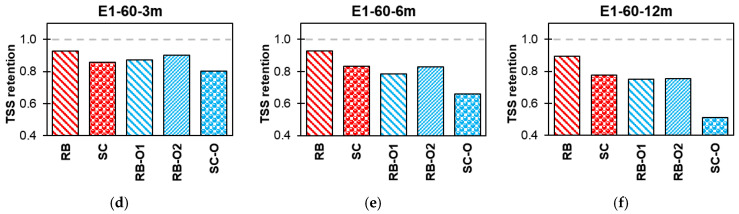
Effect of E1 (alkaline) TSS retention. (**a**) Lab-exposed for 3 months. (**b**) Lab-exposed for 6 months. (**c**) Lab-exposed for 12 months. (**d**) Oven-exposed for 3 months. (**e**) Oven-exposed for 6 months. (**f**) Oven-exposed for 12 months.

**Figure 16:**
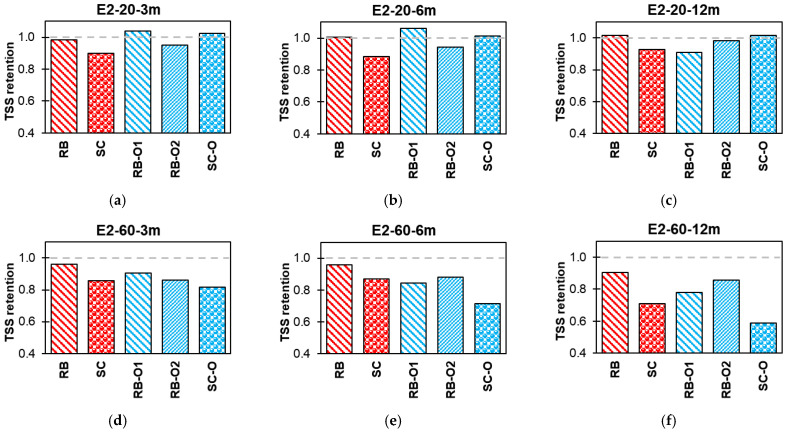
Effect of E2 (alkaline and salt) TSS retention. (**a**) Lab-exposed for 3 months. (**b**) Lab-exposed for 6 months. (**c**) Lab-exposed for 12 months. (**d**) Oven-exposed for 3 months. (**e**) Oven-exposed for 6 months. (**f**) Oven-exposed for 12 months.

**Figure 17:**
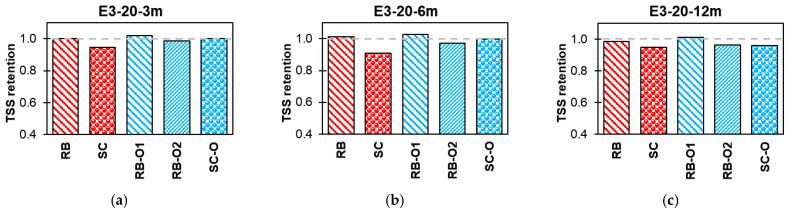

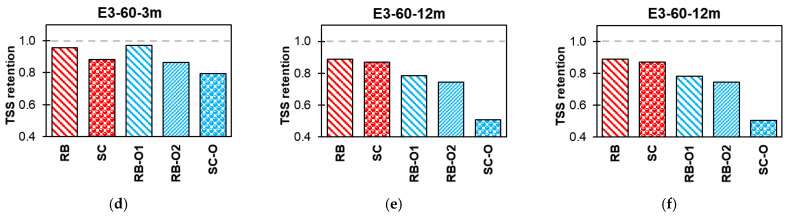
Effect of E3 (acid) TSS retention. (**a**) Lab-exposed for 3 months. (**b**) Lab-exposed for 6 months. (**c**) Lab-exposed for 12 months. (**d**) Oven-exposed for 3 months. (**e**) Oven-exposed for 6 months. (**f**) Oven-exposed for 12 months.

**Figure 18:**
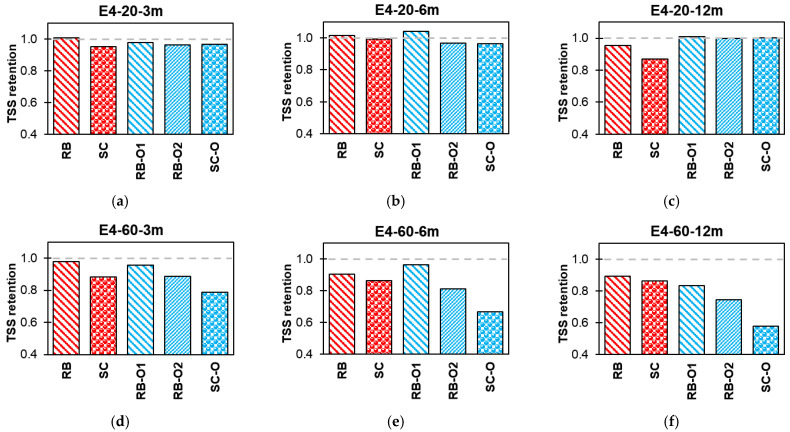
Effect of E4 (water) TSS retention. (**a**) Lab-exposed for 3 months. (**b**) Lab-exposed for 6 months. (**c**) Lab-exposed for 12 months. (**d**) Oven-exposed for 3 months. (**e**) Oven-exposed for 6 months. (**f**) Oven-exposed for 12 months.

Figure 15, Figure 16, Figure 17 and Figure 18 summarize the effects of alkaline, alkaline with salt, acid, and water solutions on the TSS retention of RB and SC new-generation bars, along with RB-O1, RB-O2, and SC-O older-generation bars. The older generation of GFRP bars exhibited good TSS retention at ambient laboratory temperature over an exposure duration of 12 months. In most cases, the older-generation bars performed better than did the new-generation sand-coated bars. However, at higher temperatures, notable reductions in TSS were observed in the older bars. The sand-coated SC-O bars exhibited a significant reduction in strength after 12 months of exposure. Hence, this comparison validates the advancements in the durability of the new-generation bars.

## 4. Transverse Shear Strength Prediction Model

### 4.1. Dataset

A dataset comprising the TSS test results for the two types of new-generation GFRP bars (RB and SC) and the three types of older-generation GFRP bars (RB-O1, RB-O2, and SC) subjected to various exposure conditions was used to develop linear regression and ANN models. The selected predictor variables include the exposure solution type (E1, E2, E3, and E4), exposure temperatures of 20 °C and 60 °C, and bar types (RB, SC, RB-O1, RB-O2, and SC-O) with diameters of 13.71, 13.02, 13.04, 13.02, and 13.22 mm, respectively. The response variable TSS ranged from 90.6 to 200.6 MPa. Categorical data, such as exposure and bar type, were entered using dummy variables to represent the data in binary form (0 or 1).

The coefficient of determination (*R*^2^) was used as a statistical metric to assess the performance of the linear regression and ANN models. The *R*^2^ values ranged from 0 to 1. A high *R*^2^ value (close to 1) indicates better model performance.

### 4.2. Linear Regression

**Figure 19:**
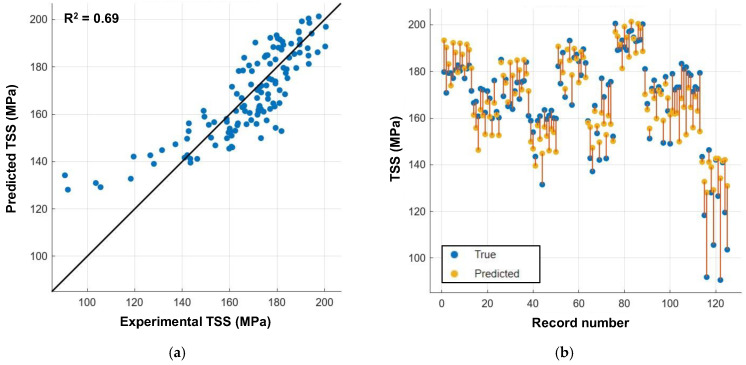
Linear regression model. (**a**) Actual versus predicted responses from the multiple linear regression model. (**b**) Response plot: variation between experimental and predicted TSS values.

Figure 19a presents a parity plot that shows the actual versus predicted values from the linear regression model. The model overestimates the TSS values at lower strengths, particularly up to 140 MPa. The linear regression model exhibited an *R*^2^ value of 0.69, indicating a low reliability. The response plot in Figure 19b shows the variations in the experimentally predicted TSS values for each input used in the model.

### 4.3. ANN Model

**Figure 20:**
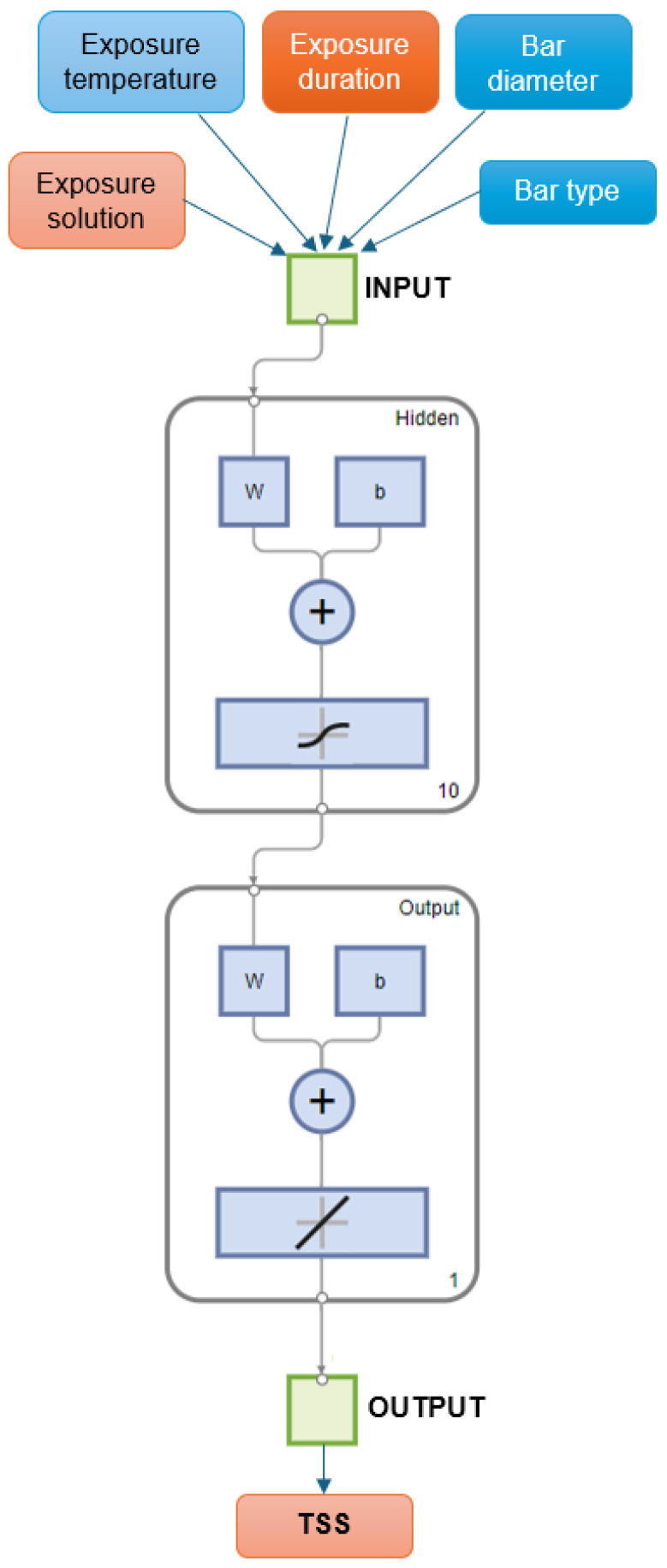
Structure of the ANN implemented in MATLAB. (Note: *w* represents weights, and *b* represents biases).

**Figure 21:**
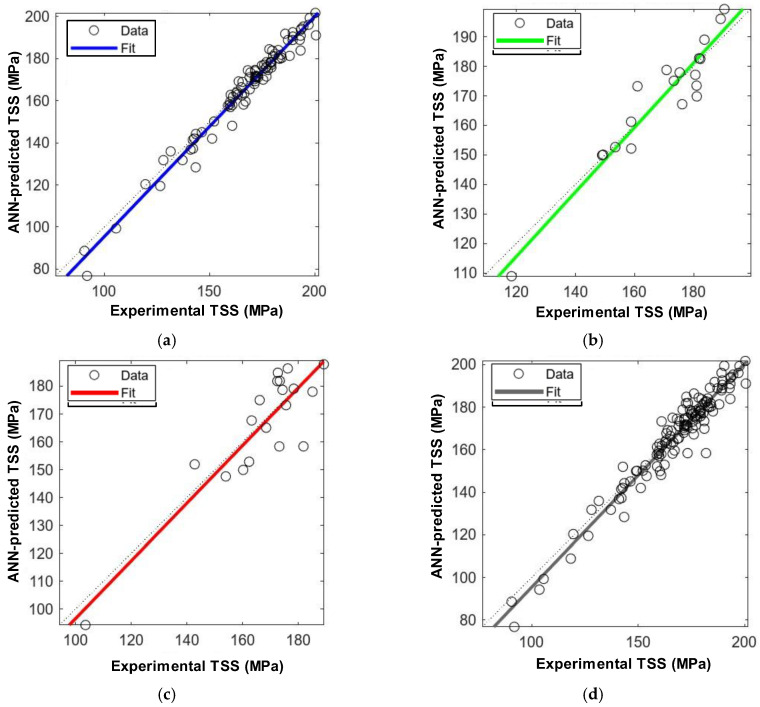
Parity plots: Experimental versus ANN predictions of ILSS. (**a**) Training subset (*R*^2^ = 0.97). (**b**) Validation subset (*R*^2^ = 0.90). (**c**) Testing subset (*R*^2^ = 0.80). (**d**) All data (*R*^2^ = 0.94).

**Figure 22:**
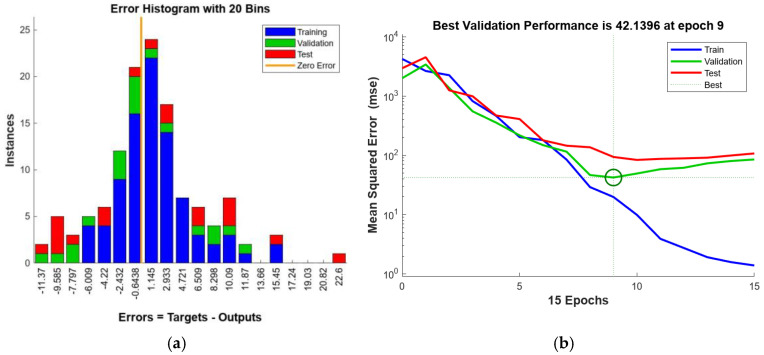
Error metrics: (**a**) Frequency histogram of the error distribution with 20 bins; (**b**) mean squared error (MSE) trends as a function of epochs for the training, validation, and test sets.

The neural network structure developed in this study, as shown in Figure 20, consists of a two-layer feed-forward network, with ten hidden layers that employ a sigmoidal transfer function as its activation function, as well as a single output layer that uses a linear transfer function. Figure 21 presents an assessment of the performance of the ANN model by using parity plots to demonstrate that the neural networks are effective in predicting the interlaminar shear strength (ILSS) of GFRP bars. For the training, validation, and testing subsets, the ANN model achieved *R*^2^ values of 0.97, 0.90, and 0.80 for the training, validation, and testing subsets, respectively. Figure 22a presents a frequency histogram with 20 bins that displays the error distribution between the target (experimental ILSS) and output (predicted ILSS) values from the training, validation, and testing subsets. In addition, Figure 22b plots the error against the number of epochs (iterations) for training, validation, and testing. In general, the mean square error (MSE) decreases up to a certain number of epochs, and then increases as the network begins to overfit the training data. By default, the training was halted after six consecutive increases in the validation MSE, and the best performance was selected from the epoch with the lowest error. In this study, the optimal validation performance was achieved at an MSE of 42.14 in the ninth epoch.

These results indicate that the ANN can be used as a reliable and effective tool for predicting mechanical properties, such as the TSS values of GFRP bars. The superior performance of the ANN, compared with the linear regression model, is attributable to its ability to generate nonlinear relationships between the input and output variables.

## 5. Conclusions

In this study, the durability of GFRP bars with ribbed and sand-coated surface textures was investigated after exposure to four solutions (E1: alkaline; E2: alkaline and salt; E3: acidic; and E4: water), two different temperature levels (ambient laboratory and high-temperature conditions), and three exposure durations (3, 6, and 12 months). The following conclusions were drawn from the research:

- The transverse shear strength retention of the bars identified in the experimental program revealed that the ribbed GFRP bars exhibited good resilience under ambient laboratory conditions, with no notable reduction in strength after 12 months; whereas a dip in strength occurred under high-temperature conditions. After 12 months at 60 °C, RB bars exhibited TSS reductions of 10.6%, 9.7%, 11.1%, and 10.9% owing to exposure solutions E1, E2, E3, and E4, respectively. The sand-coated GFRP exhibited slight reductions in strength under ambient laboratory conditions, with moderate reductions in strength under high-temperature conditions, particularly after 12 months. After 12 months at 60 °C, SC bars exhibited TSS reductions of 22.5%, 29.0%, 13.0%, 13.7% owing to exposure solutions E1, E2, E3, and E4, respectively. The mechanical strength deterioration of GFRP bars in harsh environments is generally due to resin matrix hydrolysis and glass fiber leaching. Hydrolysis breaks polymer chains, weakening molecular bonds, while leaching degrades the glass structure, forming a gel that accelerates water and alkali transport, leading to swelling and microcracking at the glass-matrix interface.
- Three types of older generation bars, including two types of ribbed (RB-O1 and RB-O2) and sand-coated (SC-O) GFRP bars, were compared to understand the generational advancement in the durability performance of GFRP bars. All three types of older generation bars, particularly the sand-coated bars, exhibited severe reductions in transverse shear strength, in contrast to the reasonably good performance of the new-generation bars.
- Prediction models based on linear regression and artificial neural networks were generated and compared. The coefficient of determination of the ANN prediction model (*R*^2^ = 0.94) was found to be significantly higher than that of the multiple linear regression model (*R*^2^ = 0.69), owing to its ability to consider the nonlinearity of the strength retention in the exposed GFRP bars.

## Figures and Tables

**Table 1:** GFRP bar properties.

Bar Type	Ribbed Type (RB)	Sand-Coated Type (SC)
Surface texture	Ribbed	Sand-coated
Diameter (mm, immersion test)	13.71 ± 0.049	13.02 ± 0.083
Glass fiber type	Electrical glass	Electrical glass
Matrix type	Vinyl ester resin	Vinyl ester resin
24 h moisture uptake (%)	0.019 ± 0.0007	0.027 ± 0.0007
Ultimate tensile strength (MPa)	956.4 ± 18.5	1029.6 ± 27.5
Ultimate strain (%)	~1.9%	~2.4%
Modulus of elasticity (GPa)	49.5 ± 0.96	43.6 ± 3.32
Transverse shear strength (MPa)	180.1 ± 3.1	185.1 ± 10.2

**Table 2:** Detailed test matrix in tabular format.

Conditioning Solution	Duration (Months)	Temperature	Name
E1 (alkaline)	3	20 °C	E1-20-3
	6		E1-20-6
	12		E1-20-12
	3	60 °C	E1-60-3
	6		E1-60-6
	12		E1-60-12
E2 (alkaline and salt)	3	20 °C	E2-20-3
	6		E2-20-6
	12		E2-20-12
	3	60 °C	E2-60-3
	6		E2-60-6
	12		E2-60-12
E3 (acid)	3	20 °C	E3-20-3
	6		E3-20-6
	12		E3-20-12
	3	60 °C	E3-60-3
	6		E3-60-6
	12		E3-60-12
E4 (water)	3	20 °C	E4-20-3
	6		E4-20-6
	12		E4-20-12
	3	60 °C	E4-60-3
	6		E4-60-6
	12		E4-60-12

**Table 3:** GFRP (older-generation) bar properties [15].

Bar Type	Ribbed Type (RB-O1)	Ribbed Type (RB-O2)	Sand-Coated Type (SC-O)
Surface texture	Ribbed	Ribbed	Sand-coated
Diameter (mm) (immersion test)	13.04	13.02	13.22
Glass fiber type	Electrical glass	Electrical glass	Electrical CR-glass
Matrix type	Vinyl ester resin	Urethane-modified vinyl ester resin	Vinyl ester resin
24h moisture uptake (%)	0.046	0.055	0.092
Transverse shear strength (MPa) [15]	180.27	200.56	179.01

## Data Availability

The original contributions presented in the study are included in the article, further inquiries can be directed to the author.
